# Etymologia: Peste des Petits Ruminants

**DOI:** 10.3201/eid2012.ET2012

**Published:** 2014-12

**Keywords:** etymologia, peste des petits ruminants, peste des petits ruminants virus, viruses, morbillivirus, ovine rinderpest, goat plague

## Peste des petits ruminants [pest dā pə-teʹ ru-me-nahʹ]

From the French for “plague of the small, hooved mammals,” peste des petits ruminants (PPR) is a severe (mortality rate may be >90%), highly contagious disease of sheep and goats. PPR was first described in Côte d’Ivoire in 1942 and soon discovered in other countries in West Africa. In more recent decades, it has spread to many other parts of the world, including East Africa, the Middle East, and Asia. Also known as ovine rinderpest and goat plague, PPR is caused by a morbillivirus closely related to the rinderpest virus of cattle and buffaloes (which was eradicated in 2011) and the measles virus of humans.
